# Neurophysiological signatures of latent brain vulnerability in perioperative neurocognitive disorders: integrating EEG, cerebral oximetry, and processed EEG

**DOI:** 10.3389/fmed.2026.1941017

**Published:** 2026-09-22

**Authors:** Yuhan Meng, Ruyi Xia, Jiahai Ma

**Affiliations:** 1 Second Clinical Medical College, Shandong Medical and Pharmaceutical University, Yantai, Shandong, China; 2 School of Anesthesiology, Shandong Second Medical University, Weifang, Shandong, China; 3 Department of Anesthesiology, Yantai Yuhuangding Hospital, Yantai, Shandong, China

**Keywords:** brain vulnerability, cerebral oximetry, electroencephalography, near-infrared spectroscopy, perioperative neurocognitive disorders, postoperative delirium, processed EEG, risk stratification

## Abstract

Perioperative neurocognitive disorders (PND) remain difficult to prevent because conventional cognitive assessments may fail to capture brain vulnerability in patients who appear cognitively normal before anesthesia and surgery. This narrative review examines the potential role of objective perioperative neuromonitoring markers as complements to cognitive scales, focusing on electroencephalography (EEG), near-infrared spectroscopy (NIRS)/cerebral oximetry, and processed EEG/bispectral index (BIS). Evidence from perioperative studies and related neurophysiological literature suggests that reduced or slowed alpha activity, lower spectral edge frequency, altered theta-band activity, burst suppression, cerebral oxygen desaturation burden, rSO₂ variability, impaired autoregulatory responses, and prolonged deep anesthetic exposure may reflect different dimensions of perioperative brain vulnerability. EEG primarily provides information on cortical oscillatory dynamics, anesthetic sensitivity, and network recovery; NIRS/cerebral oximetry reflects oxygen delivery, perfusion–metabolism mismatch, and hemodynamic instability; and processed EEG/BIS offers a simplified index of anesthetic-depth exposure rather than an independent neurophysiological modality equivalent to raw EEG or NIRS. Their combined value therefore depends not on simple multimodal aggregation, but on integrated interpretation across baseline assessment, anesthetic induction, intraoperative maintenance, emergence, and early postoperative recovery. Current evidence is limited by heterogeneous PND definitions, device-dependent signal processing, baseline variability, and a lack of prospective trials testing monitor-guided interventions. As a narrative review without formal risk-of-bias grading, this synthesis is also vulnerable to study-selection bias. Accordingly, EEG, NIRS/cerebral oximetry, and processed EEG/BIS should be viewed as candidate objective markers for risk stratification and perioperative brain-vulnerability phenotyping, rather than as a validated early-warning system. Future studies should determine whether these signals can identify actionable vulnerability profiles beyond cognitive screening and guide individualized perioperative brain-protection strategies.

## 1 Introduction

### 1.1 Definition, clinical burden, and unmet needs of perioperative neurocognitive disorders

Perioperative neurocognitive disorders (PND) encompass a spectrum of cognitive disturbances temporally associated with surgery, anesthesia, and perioperative stress. Clinically, this spectrum includes postoperative delirium (POD), delayed neurocognitive recovery (DNR), postoperative neurocognitive disorder, and the historically used term postoperative cognitive dysfunction (POCD) (1). Current diagnostic approaches are largely based on the neurocognitive disorder framework of the fifth edition of the Diagnostic and Statistical Manual of Mental Disorders (DSM-5), supplemented by neuropsychological screening and assessment tools such as the Montreal Cognitive Assessment (MoCA) and the Mini-Mental State Examination (MMSE) (2, 3). However, these instruments primarily identify overt cognitive impairment or postoperative cognitive change, and may not adequately capture brain vulnerability in patients who appear cognitively normal before anesthesia and surgery. Other factors, including hyperlipidemia, diabetes, inflammatory status, lower educational attainment, and reduced preoperative cognitive reserve, have also been associated with PND (4, 5). Therefore, this review conceptualizes PND not merely as a postoperative diagnostic label, but as a dynamic perioperative process involving preoperative vulnerability, intraoperative neurophysiological and hemodynamic exposure, and postoperative cognitive outcomes. Rather than presenting EEG, NIRS, and processed EEG as a validated standalone prediction system, we aim to critically evaluate their potential roles as candidate objective markers of brain vulnerability beyond conventional cognitive scales. Sleep-related factors, pain, and other perioperative neuropsychiatric symptoms are discussed only when they serve as potential risk factors, comorbidities, or mediators of PND/POD risk, rather than as independent outcomes of this review.

### 1.2 Pathophysiological basis of PND relevant to multimodal monitoring

PND is unlikely to be caused by a single factor. Rather, it more plausibly arises from the interaction between baseline cerebral vulnerability and perioperative stress. In older patients, patients with chronic comorbidities, and those with reduced preoperative cognitive reserve, cerebrovascular microembolism, cerebral hypoperfusion, neuroinflammation, and anesthesia-related EEG alterations may jointly induce neuronal dysfunction (6). From the perspective of multimodal monitoring, the processes most relevant to risk stratification include cerebral oxygen supply-demand imbalance, impaired neurovascular coupling, alterations in specific EEG oscillatory bands, and anesthetic-depth-related EEG suppression. Neuroinflammation, the gut-brain axis, metabolic homeostasis, and genetic or epigenetic mechanisms provide important pathological substrates for these functional signal changes.

The mechanistic discussion below is therefore limited to whether a pathway has a plausible counterpart in EEG or cerebral-oxygenation signals; it does not imply that routine monitors directly diagnose inflammation, microbiome changes, cellular senescence, or ferroptosis.

#### 1.2.1 Neuroinflammatory pathways

Surgical trauma and related stressors can promote the release of damage-associated molecular patterns such as HMGB1 and increase blood-brain barrier permeability. Subsequently, microglia and astrocytes are activated, and the release of proinflammatory cytokines such as IL-1β, IL-6, and TNF-α increases, thereby contributing to the development and progression of PND. Existing studies suggest that IL-6 plays a key role in perioperative inflammatory responses and may represent a potential target for modulating PND-related neuroinflammation (7).

#### 1.2.2 Gut-brain axis and microbial regulation

The gut microbiota may also influence the occurrence of PND through the gut-brain axis. Anesthesia and surgery can induce gut microbiome dysbiosis and regulate the C3/C3aR pathway in the central nervous system (CNS) through the microbiota-gut-brain axis. This pathway participates in early neuroinflammatory interactions between astrocytes and microglia and may amplify neuroinflammatory responses (8, 9).

#### 1.2.3 Metabolic and cellular homeostatic disturbances

During general anesthesia, cerebral metabolic demand and activity remain highly relevant. Dysregulated glucose metabolism, aberrant activation of glycolysis, and lactate accumulation may induce oxidative stress and synaptic dysfunction, further increasing PND risk. Different anesthetic agents may exert distinct effects on metabolic pathways. For example, propofol, an intravenous anesthetic, and the inhalational anesthetics sevoflurane and isoflurane may differ in their metabolic regulation and associations with PND (10–12).

#### 1.2.4 Emerging cross-system mechanisms

The lung-brain axis has recently attracted attention in PND mechanistic research. Inhalational anesthesia and mechanical ventilation may promote beta-amyloid deposition and blood-brain barrier injury through pulmonary microbial imbalance and lung-derived inflammatory responses (13, 14). These physiological domains, monitoring outputs, limitations, and evidence positions are summarized in Table 1.

**Table 1:** Physiological domains, monitoring outputs, limitations, and evidence positioning for perioperative brain-vulnerability research.

Monitoring layer/domain	Direct physiological target	Representative outputs	Practical utility	Main limitations	Evidence position for PND
Raw EEG	Cortical electrical activity and network dynamics	Alpha/theta/beta power; peak alpha frequency; SEF; aperiodic activity; connectivity; suppression	Waveform and spectrogram interpretation; rapid drug-response and state-transition assessment	Artifact, montage/reference, age, agent/dose, temperature, and analysis-window dependence	Multiple direct associations, mostly observational; thresholds and incremental prediction remain unvalidated
NIRS/cerebral oximetry	Regional frontal oxygenation and hemodynamic balance	rSO₂ level and desaturation burden; HbO/HbR; MAP-rSO₂ relationships	Continuous bedside detection of oxygen-delivery or perfusion imbalance	Extracranial contamination; limited frontal sampling; baseline and inter-device variability	Direct associations in selected settings; outcome benefit of threshold-guided intervention is inconsistent
Processed EEG: BIS, PSI/SedLine, Entropy, Narcotrend	Derived frontal EEG representation of hypnotic exposure and suppression—not an independent signal source	Platform-specific index; suppression ratio; trend and alarm display	Rapid interpretation, titration support, documentation, and protocol adherence	Proprietary, noninterchangeable algorithms; delay and artifact/drug/age effects; cannot replace raw EEG	Pragmatic exposure/implementation layer; randomized neurocognitive outcomes are mixed and no universal target exists
Cross-modal interpretation	Concordance or mismatch between electrophysiology, oxygenation, exposure, and systemic physiology	EEG slowing with rSO₂ decline; suppression with stable rSO₂; abnormal raw EEG despite target pEEG	Helps identify plausible source of an abnormal trend and prioritize verification	Synchronization, missing data, artifact propagation, feature selection, and model interpretability	Physiologically plausible; prospective incremental value beyond clinical models is unproven
Background biology not directly captured	Inflammation, metabolic dysregulation, gut/lung-brain signaling, senescence, and ferroptosis	No specific routine EEG, NIRS, or pEEG output	Provides mechanistic context only	Cannot be inferred directly from routine neuromonitoring	Indirect evidence; should not be labeled a monitor-derived biomarker

In summary, PND has become an important issue in the perioperative management of older surgical patients, while insufficient diagnostic standardization and limited effective interventions remain major clinical challenges. With advances in mechanistic research, the pathophysiological understanding of PND has expanded from neuroinflammation to include cerebral perfusion, cerebral oxygen metabolism, neurovascular coupling, metabolic reprogramming, cellular senescence, and neural circuitry (6, 7). Some of these mechanisms may generate monitorable electrophysiological or oxygenation-related signals, whereas others remain background biological processes that are not directly captured by routine monitoring. The following sections therefore focus on two source-signal domains—raw EEG and NIRS/cerebral oximetry—plus a derived pEEG exposure layer, and discuss their evidentiary boundaries for PND risk stratification. The multi-hit mechanistic relationships and representative clinical outcomes are summarized in Figure 1.

**Figure 1:**
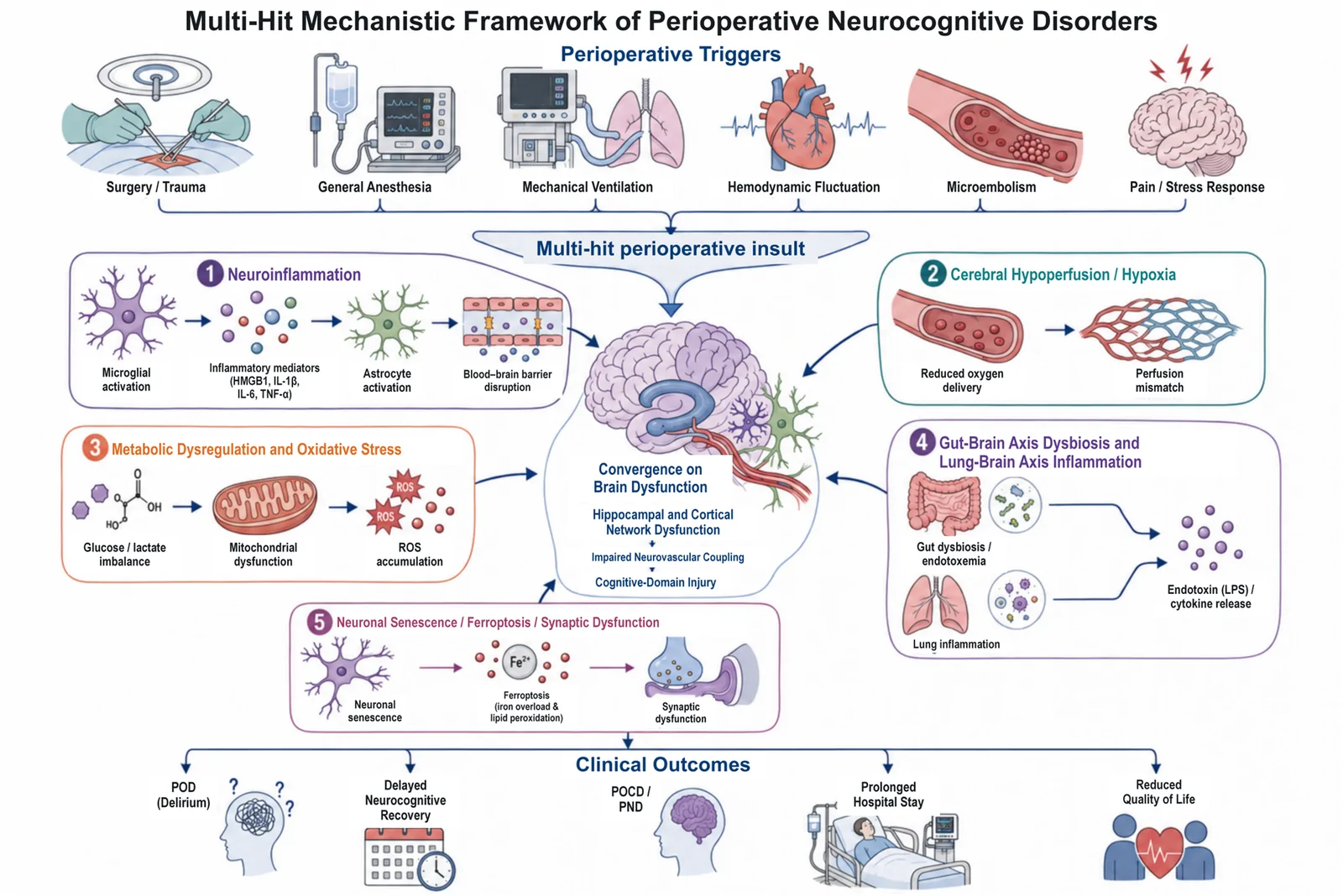
Multi-hit mechanistic framework linking perioperative insults to neurocognitive outcomes.

Perioperative triggers—including surgical trauma, general anesthesia, mechanical ventilation, hemodynamic fluctuations, microembolism, and pain/stress responses—may converge through neuroinflammation, cerebral hypoperfusion/hypoxia, metabolic dysregulation and oxidative stress, gut–brain and lung–brain inflammatory crosstalk, and neuronal senescence, ferroptosis, and synaptic dysfunction. These pathways may converge on hippocampal and cortical network dysfunction, impaired neurovascular coupling, and domain-specific cognitive injury, with clinical manifestations including postoperative delirium (POD), delayed neurocognitive recovery (DNR), postoperative cognitive dysfunction (POCD), prolonged hospitalization, and reduced quality of life. The schematic summarizes mechanistic plausibility and does not imply that routine perioperative monitors directly measure all pathways.

### 1.3 Narrative review methods

Because this article is a narrative review, we performed a focused literature search rather than a systematic-review workflow. PubMed/MEDLINE and the Cochrane Library were searched from database inception to 20 August 2026 (the date of the final search update) using combinations of terms for PND, POD, DNR, or POCD with EEG, processed EEG or anesthetic depth, cerebral oximetry or NIRS, neurovascular coupling, multimodal monitoring, and machine learning. Reference lists of key guidelines, randomized trials, and recent reviews were also examined.

Adult perioperative studies were prioritized, with randomized trials, prospective cohorts, systematic reviews or meta-analyses, and professional guidance given greatest weight. Non-perioperative and mechanistic studies were used only to contextualize physiology or technical feasibility. Selection was guided by relevance, directness, study design, and recency. Consistent with a narrative review, no PRISMA flow diagram, duplicate independent screening, formal risk-of-bias instrument, or GRADE assessment was undertaken; the evidence hierarchy in Table 2 is therefore pragmatic rather than formal.

**Table 2:** Pragmatic evidence hierarchy for objective neuromonitoring markers in PND research.

Evidence level	Evidence source	Use in this review
Direct clinical evidence	Perioperative POD/PND/DNR studies using EEG, NIRS/cerebral oximetry, or pEEG	Main basis for candidate risk markers
Supportive perioperative evidence	Studies of anesthetic depth, cerebral oxygenation, autoregulation, or EEG physiology without PND endpoints	Supports physiological plausibility
Indirect mechanistic evidence	AD, MCI, acute brain injury, cognitive-task, or neurovascular-coupling studies	Mechanistic or technical reference only
Technical/AI evidence	Signal fusion, BCI, machine-learning, and time-series modeling studies	Feasibility evidence; requires perioperative validation

## 2 Complementary value of EEG, NIRS/cerebral oximetry, and processed EEG monitoring

### 2.1 Independent physiological signals and the derived processed-EEG layer

The framework is organized around two independent physiological signal domains: cortical electrophysiology from raw EEG and regional cerebral oxygenation/hemodynamics from NIRS/cerebral oximetry. Processed-EEG (pEEG) indices are transformations of frontal EEG rather than a third independent physiological modality. We therefore treat pEEG as a derived exposure and implementation layer that can summarize hypnotic-state trends and suppression, while raw waveforms or spectrograms remain necessary for physiological interpretation. In this review, multimodal synthesis refers principally to EEG-NIRS complementarity, with pEEG used to operationalize anesthetic exposure in routine care and research protocols. EEG is sensitive to level of consciousness, anesthetic depth, cerebral hypoperfusion, ischemic events, and epileptiform discharges. Contemporary European guidance discusses processed-EEG and raw-EEG interpretation within multicomponent delirium prevention, but does not establish a universal index target or a validated EEG-guided strategy for preventing PND (15, 16). Perioperative reviews likewise emphasize the complementary interpretation of EEG and cerebral oxygenation monitoring (17).

pEEG is used here as a class term rather than as a synonym for BIS. Commercial platforms include BIS, the Patient State Index (PSI; SedLine), State and Response Entropy, and Narcotrend; their proprietary feature sets, numerical scales, smoothing, artifact rejection, and suppression algorithms differ, so numerical values are not interchangeable (18, 19). When raw EEG is already available, pEEG adds convenience rather than independent physiology: a continuously displayed numerical trend and suppression metric can facilitate rapid recognition, titration, documentation, and protocol adherence where expert raw-EEG interpretation is unavailable. This practical value must be weighed against processing delay, electromyographic or electrocautery contamination, age and drug effects, neuromuscular blockade, and cross-device discordance. Accordingly, pEEG should be interpreted with the device's signal-quality information and the underlying waveform or spectrogram, not used as a stand-alone cortical-state measure (18, 19). Practical implementation also requires clinician training in EEG and pEEG interpretation (20).

### 2.2 Clinical need for objective biomarkers and evidence gaps

Existing studies have reported several single monitoring indices with predictive value. For example, reduced intraoperative alpha power (AUC=0.73), decreased peak alpha frequency (AUC=0.83), and lower cerebral oxygen saturation [68 vs. 72 (4), *p* = 0.011] have been associated with DNR (21). Alpha/beta power reduction one minute after anesthetic induction and a decreased aperiodic offset at 15 min were associated with postoperative delirium in a development model (AUC=0.73) (22). Increased frontal-parietal theta connectivity has also been associated with postoperative delirium (*P* = 0.034). However, most of these findings come from single-center, small-sample, or observational studies, and some conclusions are inconsistent. For example, one study did not find a significant association between preoperative alpha power and postoperative cognitive scores (21). Evaluation of multimodal biomarkers therefore requires evidence stratification: direct perioperative evidence from PND/POD/POCD populations should serve as the core basis; perioperative EEG, cerebral oxygenation, and anesthetic-depth studies can provide supportive evidence; and EEG-fNIRS studies in Alzheimer disease, mild cognitive impairment, acute brain injury, and brain-computer interfaces are more appropriate as mechanistic and technical feasibility references, rather than as direct PND prediction evidence. A reliable biomarker system that fuses multimodal physiological signals and dynamically identifies high-risk patients intraoperatively or during early emergence remains lacking. Further prospective analysis of raw EEG-NIRS relationships, with pEEG exposure and clinical variables treated explicitly, is needed before claiming incremental prediction or earlier perioperative brain protection (23, 24). Importantly, the literature also includes negative and inconclusive findings. Several perioperative studies have failed to demonstrate consistent clinical benefits of neuromonitoring-guided interventions for postoperative delirium or neurocognitive outcomes. For example, EEG-guided anesthetic administration reduced anesthetic exposure and EEG suppression in the ENGAGES trial, but did not significantly reduce the incidence of postoperative delirium compared with usual care (25). Similarly, cerebral oximetry-guided strategies have shown variable effects across surgical populations, with some analyses suggesting benefit in selected non-cardiac cohorts but less consistent effects in cardiac surgery (26). These findings indicate that abnormal EEG or NIRS signals should not be interpreted as validated intervention-triggering criteria. Rather, they should be regarded as candidate markers that require context-specific interpretation, external validation, and prospective testing of signal-guided interventions.

These associations are hypothesis-generating rather than causal. Age, baseline cognition, anesthetic agent and dose, hypotension, temperature, and surgical complexity may affect both monitored signals and neurocognitive outcomes. No prospective study has shown that an EEG-NIRS-pEEG model adds clinically meaningful prediction beyond a well-specified clinical model, and no signal-guided multimodal algorithm has demonstrated improved PND outcomes.

### 2.3 Physiological interpretive framework for multimodal complementarity

When combined, the two modalities can simultaneously provide high-temporal-resolution neural electrical activity and regional hemodynamic information (27). For example, in motor-cognitive dual-task experiments, simultaneous EEG and fNIRS recording allows extraction of task-related electrophysiological and hemodynamic components, respectively (28). Mechanistically, EEG reflects neuronal electrical activity, whereas NIRS reflects blood-flow and metabolic responses; joint analysis can therefore help identify abnormal neurovascular coupling (29). In assessments of cerebrovascular autoregulation, combining NIRS with raw EEG may help relate abnormal electrical patterns, such as burst suppression, to hemodynamic imbalance, while pEEG provides only a derived exposure context (30). Because pEEG is susceptible to electromyographic interference, drug effects, and algorithmic compression, any apparent relationship with NIRS should be checked against the underlying EEG before physiological attribution (31). To move beyond parallel descriptions of individual modalities, combined EEG, NIRS/cerebral oximetry, and processed EEG/BIS signals should be interpreted according to their concordant or discordant patterns. Table 3 summarizes how representative patterns can be verified, contextualized, and linked to evidence-bounded bedside responses. Studies using simultaneous EEG-fNIRS have quantified hemodynamic correlates of spontaneous neural activity, while reviews of fNIRS emphasize that these measures are indirect and method-dependent (32, 33). The landmark randomized trials and meta-analyses evaluating EEG- or pEEG-guided anesthetic management and postoperative neurocognitive outcomes are summarized in Table 4.

**Table 3:** Evidence-bounded interpretation and bedside response to representative monitoring patterns.

Signal pattern	Immediate verification	Reversible causes or context	Reasonable bedside response	PND-specific evidence boundary
Low or absent alpha with stable rSO₂	Check electrodes/artifact and inspect raw EEG or spectrogram; compare with age/baseline	Age; baseline cognition; agent and concentration; temperature; neuromuscular activity	If exposure appears excessive and conditions permit, consider cautious titration; otherwise document and contextualize	Association marker only; no validated alpha target or proof that increasing alpha prevents PND
EEG slowing with sustained rSO₂ decline	Confirm both signals, temporal alignment, probe position, and baseline	Hypotension; hypoxemia; PaCO₂; anemia; low cardiac output; head position; surgical perfusion factors	Correct conventional reversible causes and reassess both modalities	Physiologically plausible mismatch; no outcome-validated combined trigger for PND prevention
Burst suppression with stable rSO₂	Confirm suppression on raw EEG and exclude artifact	Hypnotic exposure; hypothermia; metabolic disturbance; baseline vulnerability	Review anesthetic dose and other causes; reduce exposure when clinically appropriate without compromising anesthesia	Reducing suppression has not consistently reduced POD in randomized trials
Sustained rSO₂ decline with relatively preserved EEG	Check probe contact, individualized baseline, extracranial contamination, and device context	SpO₂; MAP; PaCO₂; hemoglobin; cardiac output; venous obstruction; surgical factors	Correct conventional oxygen-delivery/perfusion abnormalities and reassess trend	Absolute and relative thresholds vary; no universal PND-prevention threshold is validated
Target-range pEEG but abnormal raw EEG	Check device signal quality, waveform/spectrogram, EMG, electrocautery, and processing delay	Agent-specific signature; neuromuscular blockade; proprietary algorithm behavior	Interpret the raw EEG and clinical state; do not titrate to the index alone	pEEG values are platform-specific and are not an independent brain-vulnerability biomarker

**Table 4:** Landmark randomized trials and meta-analyses of EEG- or pEEG-guided anesthetic management and postoperative neurocognitive outcomes.

Study/population	Intervention or contrast	Main neurocognitive result	Critical interpretation
CODA (77); 921 older adults, major noncardiac surgery	BIS-guided anesthesia versus blinded routine care	POD 15.6% vs. 24.1%; 3-month POCD 10.2% vs. 14.7%	Supports possible benefit in this setting, but does not establish a universal pEEG target
Radtke et al. (78); 1,155 analyzed patients	Depth monitoring versus blinded monitoring	POD 16.7% vs. 21.4%; no POCD reduction at 7 days or 3 months	Effect differed by outcome and did not extend to postoperative cognitive testing
STRIDE (79); 200 older hip-fracture patients	Lighter versus heavier propofol sedation during spinal anesthesia	POD 34% vs. 39%; no significant overall benefit	Not a general-anesthesia pEEG trial; subgroup finding is hypothesis-generating
ENGAGES (25); 1,232 older adults, major surgery	EEG-guided anesthetic administration versus usual care	POD 26.0% vs. 23.0% despite lower MAC and less EEG suppression	Clear exposure modification without clinical benefit challenges simple causality
ADAPT-2 (80); 223 older adults, major noncardiac surgery	Algorithm intended to reduce EEG suppression versus blinded monitoring	POD 17% vs. 20%; not statistically significant	Reducing a physiological exposure did not demonstrate improved delirium outcome
BALANCED delirium substudy (81); 515 analyzable patients	Lighter BIS target 50 versus deeper target 35	POD 19% vs. 28%; OR 0.58	Positive substudy; population, achieved separation, and co-interventions affect generalizability
ENGAGES-Canada (82); 1,140 cardiac-surgery patients	EEG-guided anesthetic administration versus usual care	POD 18.15% vs. 18.10%	No benefit despite lower anesthetic administration and suppression
Meta-analyses (83, 84)	2023: 9 RCTs/4,648 patients; 2025: 12 RCTs/4,523 patients	2023 OR 0.78 (*p* = 0.054); 2025 RR 0.81 (95% CI 0.69-0.95)	Suggests possible modest average benefit, with clinical and regional heterogeneity

### 2.4 Levels of multimodal fusion from synchronous acquisition to risk stratification

From the perspective of risk-model development, EEG-NIRS integration with derived pEEG exposure data can be divided into three levels. The first is the synchronous acquisition level, in which raw EEG, NIRS oxygenation signals, and platform-specific pEEG values are recorded simultaneously during anesthetic induction, intraoperative maintenance, and early emergence, with traceable timestamps and sensor locations. The second is the feature-fusion level, in which alpha/theta/beta band powers, functional connectivity, SEF, burst suppression, the magnitude and duration of rSO₂ decline, HbO/HbR changes, and pEEG exposure trends are incorporated into a unified analytic framework. The third is the clinical decision level, in which age, preoperative cognitive status, comorbidities, anesthetic drugs, hemodynamics, and surgical type are combined to generate interpretable risk stratification. Combined EEG and NIRS monitoring has demonstrated technical feasibility in multiple settings. For example, in neonatal hypoxic-ischemic encephalopathy, EEG-NIRS partial wavelet coherence can quantify neurovascular coupling, and reduced coupling has been associated with adverse MRI outcomes; further incorporation of SEF can improve sensitivity for identifying brain injury (22). In cognitive tasks among healthy participants, EEG-NIRS co-registration can also identify multimodal signal patterns related to mental concentration (34). These studies provide methodological references for perioperative multimodal risk stratification, but their clinical extrapolation requires further validation in PND populations. EEG-informed fNIRS analysis and other feature-level methods may improve multimodal signal characterization, but they remain research approaches requiring perioperative validation (35).

The candidate objective markers, their evidence base, and translational challenges are summarized in Table 5. The corresponding monitoring domains and candidate outputs are illustrated in Figure 2.

**Table 5:** Candidate objective markers, evidence base, and translational challenges for perioperative brain-vulnerability assessment.

Candidate index	Modality	Potential pathophysiological process	Current evidence base	Translational challenge
Reduced alpha power/decreased peak alpha frequency	EEG	Reduced neural reserve and cortical rhythm vulnerability after anesthetic induction	Direct perioperative cohort evidence suggests associations with DNR or POD risk, but findings are not uniform across cohorts.	Strongly influenced by age, anesthetic drugs, and baseline cognitive status
Increased frontal-parietal theta connectivity	EEG	Abnormal functional connectivity, increased attentional-control load, or abnormal synchrony	Direct POD-associated evidence exists in selected perioperative cohorts; reproducibility remains limited.	Limited sample sizes; heterogeneity in surgical type and anesthetic regimen
Reduced SEF/burst suppression	EEG/pEEG	Excessive anesthetic depth, cortical activity suppression, and insufficient cerebral functional reserve	Direct perioperative evidence links suppression-related exposure to delirium risk, but interventional trial results remain inconsistent.	Limited comparability across devices and algorithms; thresholds not standardized
Sustained rSO₂ decline or values below individualized baseline	NIRS	Cerebral oxygen supply-demand imbalance, hypoperfusion, or impaired cerebral autoregulation	Direct perioperative outcome evidence is strongest in selected high-risk surgical settings, but cerebral oximetry-guided intervention studies show mixed results.	Absolute values are affected by interindividual variability; interventional benefit remains to be verified
Abnormal EEG-NIRS neurovascular coupling	EEG + NIRS	Mismatch between neural activity and blood-flow/metabolic response	Mainly indirect mechanistic evidence from cognitive impairment, acute brain injury, and task studies; limited direct PND evidence.	Requires standardized synchronous acquisition, artifact processing, and coupling-analysis pipelines
Multimodal machine-learning risk score (EEG + NIRS/cerebral oximetry + pEEG + clinical variables)	EEG + NIRS/cerebral oximetry + pEEG + clinical variables	Integrated reflection of cerebral vulnerability, anesthetic-depth risk, and oxygen-metabolic risk	Primarily technical feasibility evidence; prospective perioperative validation remains insufficient.	Interpretability, generalizability, and prospective interventional validation remain insufficient

**Figure 2:**
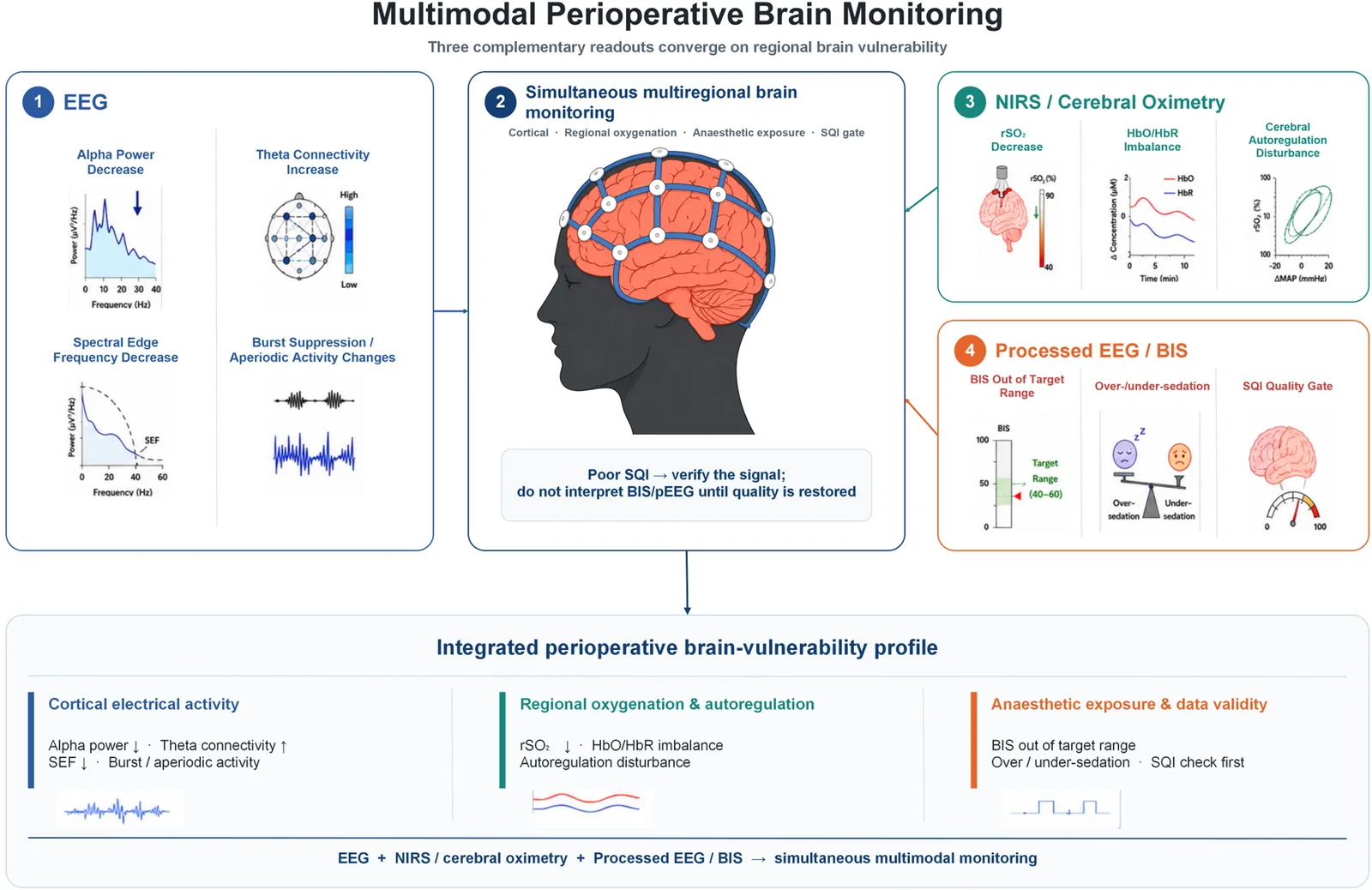
Multimodal perioperative brain monitoring with SQI as a signal-quality gate for candidate brain-vulnerability profiling in PND.

The schematic presents complementary readouts rather than three independent physiological sources. Raw EEG characterizes cortical electrical activity and oscillatory/network features; NIRS/cerebral oximetry characterizes regional oxygenation, HbO/HbR balance, and autoregulatory responses; and processed EEG/BIS provides a device-dependent index of anesthetic-depth exposure. The Signal Quality Index (SQI) is a signal-reliability measure, not a cortical-state or brain-vulnerability marker. It is used only as a quality-control gate: poor-quality segments prompt signal verification, and the accompanying BIS/pEEG value is withheld from interpretation and risk scoring until quality is restored. Synchronous acquisition across regions and perioperative stages may support integrated interpretation, but the resulting profile remains hypothesis-generating and is not a validated PND prediction or treatment algorithm.

## 3 Data acquisition, synchronization, and standardization: From technical feasibility to clinical usability

### 3.1 Coordinated acquisition of EEG, NIRS/cerebral oximetry, and processed EEG signals

Electroencephalography (EEG) records the summated postsynaptic potentials of cortical neurons and reflects dynamic changes in neural electrical activity. Its major advantage is millisecond-level temporal resolution, enabling sensitive detection of changes in consciousness during anesthetic induction, cerebral ischemic events, and epileptiform discharges (36, 37). Near-infrared spectroscopy (NIRS) uses differences in the absorption of specific near-infrared wavelengths by oxyhemoglobin (HbO) and deoxyhemoglobin (HbR) to continuously and noninvasively monitor regional cerebral tissue oxygen saturation (rSO₂), thereby reflecting cerebral blood flow and oxygen metabolism (38) index replace analysis of raw EEG spectra and network features. The core objective is to synchronize the two source signals—raw EEG and NIRS/cerebral oximetry—while recording platform-specific pEEG exposure trends and clinical events on the same time axis. Studies indicate that EEG reflects the effects of anesthetic agents on cortical oscillations, whereas NIRS records corresponding neurovascular coupling changes; combined assessment enables a more complete evaluation of the matching among cerebral perfusion, metabolism, and electrical activity (29, 39). In addition, integrated systems using shared monitoring modules, miniaturized design, and wireless transmission can improve the efficiency of synchronized EEG and fNIRS acquisition and enhance the quality and feasibility of long-term monitoring (40).

### 3.2 Temporal synchronization, spatial co-registration, and feature fusion

Effective fusion of EEG and NIRS signals requires temporal and spatial synchronization. Temporal synchronization means aligning the two modalities on the same time axis, typically through hardware triggers or a shared clock, with timing accuracy matched to the planned analysis and to reduce the influence of timing offsets on neurovascular coupling estimates (29). Spatial synchronization relies on co-registered scalp sensor layouts, allowing EEG electrodes and NIRS source-detector pairs to cover the same or adjacent cortical regions and thereby increasing the likelihood that measured electrical activity and hemodynamic changes originate from the same functional brain area. Recent advances in multimodal signal-processing algorithms have improved the analytic capacity of combined EEG-NIRS monitoring. Traditional approaches often process each modality separately before late fusion, whereas newer fusion frameworks emphasize feature-level or decision-level integration to support more rapid clinical interpretation (41, 42). For example, in motor cortex-related studies, NIRS channels and EEG electrodes have been co-localized over the precentral gyrus to synchronously capture HbO dynamics and EEG time-frequency features during motor imagery tasks (43). In mental arithmetic or word-chain cognitive tasks, synchronized EEG-NIRS systems can achieve relatively high classification accuracy within short 5-second epochs, underscoring the importance of temporal-spatial synchronization for detecting task-related brain activation (44, 45). In the dynamic perioperative monitoring environment, multimodal systems must also tolerate motion artifacts and maintain spatial correspondence through real-time calibration to preserve physiological consistency between modalities (40).

### 3.3 Artifact control, baseline definition, and standardization barriers

When raw EEG and NIRS/cerebral oximetry are acquired together and pEEG trends are recorded alongside them, signal artifacts are a primary challenge. Intraoperative positioning changes, mechanical ventilation, electrosurgical equipment, and minor head motion can introduce non-neural noise and reduce the reliability of EEG and NIRS signals (29). EEG is sensitive to electromyographic activity, eye movements, and environmental electromagnetic interference; NIRS is susceptible to scalp blood-flow fluctuations, probe displacement, and optical-path obstruction (40). In older or critically ill patients, even minimal movement may destabilize the coupling between NIRS probes and the skin, leading to distorted oxygenation signals (38).

To address these issues, some studies have applied adaptive mixture independent component analysis (AMICA) and related algorithms to separate neural from non-neural components, achieving relatively high accuracy in assessing consciousness states in patients with acute brain injury (39). However, the real-time applicability of these methods in dynamic surgical environments remains limited. Multimodal monitoring also faces issues of standardization and baseline definition. Interindividual differences in anatomy, cortical thickness, skull density, and baseline brain-function status can cause substantial variability in EEG-NIRS signals, limiting the generalizability of biomarkers (46). For example, EEG spectral power features are strongly influenced by age, preoperative cognitive status, and anesthetic pharmacokinetics; older patients often exhibit increased slow-wave activity and attenuated alpha oscillations, which may obscure changes specific to neurocognitive disorders (47). NIRS-derived rSO₂ is likewise affected by cerebral blood flow, skin pigmentation, probe placement, and cranial geometry, and absolute values may differ by more than 20% across patients (48). Although some studies have proposed calibration using preoperative resting-state baselines, reliable baseline data may be difficult to obtain in emergency surgery or in patients with cognitive impairment. In addition, BIS is derived mainly from frontal EEG and is insufficiently sensitive to abnormalities in distant brain regions, limiting its ability to comprehensively reflect perioperative neurocognitive risk (47). Therefore, individualized and dynamically calibrated multimodal reference frameworks, along with standardized perioperative artifact-filtering and compensation pipelines, remain key challenges for future research (49).

Cerebral oximetry also has modality-specific limitations that should be reported explicitly. Forehead signals may be contaminated by extracranial blood flow, sample only a limited frontal cortical volume, and vary with probe position, skin and skull characteristics, hemoglobin, PaCO₂, and proprietary device algorithms (50, 51). Absolute rSO₂ values are not interchangeable across devices. Both absolute thresholds and relative declines (commonly 20% from an individualized baseline in published studies) have been used, but neither constitutes a universal, outcome-validated trigger for PND prevention; interpretation depends on the quality and timing of the baseline (50, 51). Reliability of derived fNIRS metrics likewise depends on the acquisition system and protocol, reinforcing the need for device-specific validation (52).

### 3.4 Clinical validation in cardiac surgery and other high-risk surgical settings

Cardiac surgery is often accompanied by complex hemodynamic changes and potential fluctuations in cerebral perfusion, making it a high-risk setting for PND. In recent years, studies of combined EEG and NIRS monitoring in cardiac surgery have increased, providing a basis for early identification of brain-function abnormalities using multimodal monitoring (46). EEG can capture dynamic changes in brain electrical activity during anesthetic induction, maintenance, and emergence, and can be used to assess anesthetic depth, detect ischemic events, and predict postoperative cognitive dysfunction (36, 37). NIRS provides real-time feedback by noninvasively monitoring regional cerebral oxygen saturation (rSO₂), reflecting cerebral oxygen supply-demand balance, and detecting intraoperative hypoperfusion or embolic events (53). Multiple studies indicate that combined EEG-NIRS monitoring has clinical value in cardiac surgery. For example, older patients with preoperative cognitive impairment (MoCA <25) under propofol anesthesia exhibited reduced sleep spindle power and reduced intraoperative alpha-band power, with a positive correlation between the two (r = 0.544, *p* = 0.003), suggesting that preoperative EEG features may reflect cognitive vulnerability (54). Another study found that lower intraoperative frontal alpha-band power was associated with a higher risk of postoperative delirium (adjusted OR = 0.88, 95% CI 0.81–0.96, *P* = 0.007) (55). Regarding NIRS, a prospective cohort study showed that a cumulative rSO₂ desaturation burden, defined by the magnitude and duration of rSO₂ decline greater than 20% from baseline, was significantly associated with POCD (56, 57). Overall, EEG-NIRS combined monitoring can provide multidimensional brain-function information, but whether monitoring thresholds and intervention strategies translate into clear clinical benefit remains to be verified by high-quality randomized controlled trials (58–60). A pilot CABG study in older adults also linked perioperative cerebral-oxygen changes with postoperative cognitive dysfunction, although its small sample limits inference (61).

Most signal-outcome findings in this area are observational and often derive from modest, single-center cohorts. Residual confounding, selective analytic windows, device and anesthetic heterogeneity, and inconsistent cognitive endpoints limit reproducibility and external validity. An association between a signal and PND does not establish that the signal is causal or that targeting it will improve outcomes.

## 4 Candidate biomarkers and mechanistic interpretation

### 4.1 Functional connectivity abnormalities: From network vulnerability to cognitive risk

EEG can reflect brain network activity through band power, coherence, phase synchronization, and functional connectivity, whereas NIRS/fNIRS records regional cortical oxygenation and hemodynamic responses. When combined, the two modalities can not only measure neural electrical activity itself, but also assess whether neural activity is matched by appropriate blood-flow and metabolic responses. In the perioperative setting, the frontal-parietal network is of particular interest. Frontal-parietal connectivity participates in sustained attention, task switching, and consciousness regulation, and may be especially sensitive to anesthesia and surgical stress in older patients. Studies have reported that intraoperative frontal-parietal theta connectivity is associated with postoperative delirium, whereas preoperative posterior alpha power or some resting-state indices are not consistently associated with cognitive scores (21). This suggests that PND-related signals may not arise from a single frequency band or brain region, but may instead appear as network reorganization and connectivity abnormalities within specific temporal windows. For clinical translation, functional connectivity metrics may be valuable because they can capture brain functional imbalance early. However, their limitations are also substantial: device type, electrode montage, anesthetic regimen, and analytic algorithm can all influence results, and reproducibility requires further verification. Therefore, brain network connectivity patterns may be considered candidate PND biomarkers, but at present they are better regarded as candidate risk markers rather than independent diagnostic criteria (62).

The connectivity literature remains exploratory. Most perioperative analyses use modest cohorts and differ in montage, reference scheme, frequency-band definition, connectivity estimator, and analytic window; *post hoc* feature selection and multiple comparisons may inflate apparent associations. Independent external replication is uncommon, so theta or frontoparietal connectivity should not yet be treated as a reproducible clinical threshold.

### 4.2 Impaired neurovascular coupling and cerebral oxygen-metabolic imbalance

Neurovascular coupling (NVC) refers to the physiological process by which local cerebral blood flow and oxygen metabolism are regulated in response to increased neuronal activity to meet tissue energy requirements. Combined EEG-NIRS monitoring provides a relatively direct technical route for assessing NVC. EEG reflects neuronal population activity, whereas NIRS reflects oxygen-metabolic changes such as HbO, HbR, and rSO₂. Synchronous recording allows analysis of whether changes in neural activity are accompanied by appropriate blood-flow and metabolic responses. It should be emphasized that NVC remains an exploratory PND biomarker. In patients with acute brain injury, coupling between prefrontal low-frequency HbO fluctuations and low-frequency EEG power can help distinguish consciousness states (39), but this finding cannot be directly equated with perioperative PND prediction. Key questions in PND populations include which window—anesthetic induction, intraoperative maintenance, or emergence—should be used as the primary analytic period; whether absolute rSO₂, relative decline, or duration should serve as the oxygen-metabolic index; and whether NVC thresholds should be individualized according to age, preoperative cognitive status, and surgical type. Only after prospective validation of these issues can NVC move from a mechanistic marker to a clinically usable risk-stratification tool. Multimodal EEG-fNIRS studies in mild Alzheimer disease have also reported neurovascular uncoupling, but this evidence is indirect for perioperative PND (63). Experimental work has linked NMDA-receptor overactivation with cerebral hyperemia and neurovascular injury through MEK/ERK and calcineurin/NFAT pathways, illustrating that hemodynamic abnormalities are not specific to PND (64). Neurovascular and immune mechanisms have also been implicated in delirium superimposed on dementia (65).

Direct perioperative evidence linking a quantitative EEG-NIRS neurovascular-coupling metric to PND is sparse; much of the supporting literature is mechanistic or non-perioperative. Coupling estimates are sensitive to signal quality, preprocessing, the assumed hemodynamic lag, and the selected correlation, coherence, or causal metric. No acquisition window, coupling measure, or intervention threshold is standardized for perioperative use. Recent methodological work has proposed nonlinear or multivariate approaches to quantify EEG-NIRS coupling, but these remain research methods rather than standardized perioperative indices (66).

### 4.3 Coupling between EEG oscillations and hemodynamic responses

In anesthesia and perioperative research, alpha reduction is a prominent candidate biomarker. Older patients or those with lower preoperative cognitive function are more likely to exhibit reduced alpha power, decreased peak alpha frequency, or absence of dominant alpha rhythm after anesthetic induction, which may reflect reduced responsiveness of thalamocortical networks to anesthetic agents (67, 68). Theta-band changes may be associated with postoperative delirium risk, especially when increased frontal-parietal theta connectivity indicates abnormal synchrony or compensatory network reorganization under anesthesia (21). Beta activity and aperiodic components have also gained attention because they may reflect early cortical excitability and background EEG changes during anesthetic induction (69). Compared with BIS values alone, these raw EEG features preserve more spectral information and are therefore more useful for identifying patient-specific cerebral vulnerability. However, clinical interpretation of frequency-band indices requires caution. Children and adults differ in the relationship between theta, alpha, and gamma EEG activity and hemodynamic responses during anesthesia (70), suggesting that developmental stage and cerebrovascular reactivity affect EEG-NIRS coupling. In older patients, increased slow-wave activity and reduced alpha oscillations may also reflect age-related changes rather than PND risk *per se*. Future studies should therefore avoid relying on a fixed frequency-band threshold alone and should instead integrate preoperative baseline, anesthetic drug type, hemodynamic status, and NIRS oxygenation. A more rational direction is to establish multimodal feature combinations incorporating frequency-band changes, cerebral oxygen metabolism, and clinical variables to improve the stability and interpretability of PND risk stratification.

Age and anesthetic agent are essential context for these EEG features. Frontal alpha power and peak alpha frequency generally attenuate with age, so low absolute alpha activity may reflect normal aging, baseline cognitive impairment, anesthetic concentration, or vulnerability; age-adjusted or within-person comparisons are preferable to a universal threshold (71). Propofol and volatile agents commonly produce frontal slow-alpha dynamics, whereas dexmedetomidine produces prominent slow-delta and spindle-like activity (71, 72). Ketamine may add beta-gamma activity and alternating slow/gamma-burst patterns (73), and BIS or PSI can remain relatively high during remimazolam anesthesia in some patients (74). Temperature, vigilance, dose, and analysis timing add further variability. Studies should therefore prespecify the anesthetic context and analytic window, report relative as well as absolute features, and avoid presenting alpha power, SEF, or a pEEG value as a universal cutoff.

### 4.4 Anesthetic-depth exposure, processed-EEG trends, and cognitive outcomes

Anesthetic-depth management is one of the most clinically actionable components of perioperative brain protection. Insufficient anesthesia may increase the risk of intraoperative awareness, stress responses, and hemodynamic fluctuations, whereas excessive anesthesia may lead to burst suppression, excessive suppression of brain electrical activity, and impaired cerebral perfusion regulation. BIS is widely used as a frontal EEG-derived quantitative index of anesthetic depth; however, BIS values are highly compressed and do not fully represent raw EEG spectral structure, connectivity patterns, or aperiodic components. Therefore, in PND risk assessment, BIS is better suited to assist in interpreting anesthetic-depth trends than to replace raw EEG analysis.

Existing studies suggest that patients with poorer preoperative cognitive status may show distinct EEG responses during anesthesia. In older patients undergoing coronary artery bypass grafting or valve surgery, preoperative cognitive dysfunction is associated with reduced intraoperative spectral edge frequency (SEF), but not necessarily with burst suppression duration or rSO₂ decline (75). This finding suggests that certain EEG-derived indices may reflect reduced neural reserve earlier than a single cerebral oxygenation metric. Different anesthetic agents may also change the expression of EEG biomarkers. One study found that reduced alpha power under propofol anesthesia was associated with low preoperative MoCA scores, whereas reduced theta power under sevoflurane anesthesia may have greater predictive value (76). Interpretation of EEG and BIS indices must therefore account for anesthetic drug type; otherwise, drug effects may be misinterpreted as cognitive-risk signals.

The anesthetic induction period may be a key temporal window for identifying cerebral vulnerability. Studies show that patients who develop postoperative delirium may exhibit reduced alpha and beta power as early as one minute after induction, with a reduced aperiodic offset at 15 min; models based on these parameters show some ability to predict postoperative delirium (69). This indicates that EEG abnormalities related to PND risk may appear before surgical stimulation, reflecting vulnerable responses of brain networks to anesthetic drugs and consciousness transitions. If BIS changes and NIRS-based cerebral oxygenation are monitored simultaneously during this phase, it may be possible to distinguish among drug-induced EEG suppression, hypoperfusion-related cerebral hypoxia, and abnormal responses due to reduced cerebral reserve, thereby supporting individualized anesthetic management.

Current evidence does not support simply using a specific BIS range or EEG threshold as a direct PND prediction standard. On the one hand, BIS is derived primarily from frontal EEG and is less sensitive to abnormalities in distant brain regions. On the other hand, burst suppression, reduced SEF, and decreased alpha power are all influenced by age, anesthetic drugs, temperature, metabolic status, and preoperative cognitive level. Some non-cardiac surgery studies have also failed to find stable associations between preoperative posterior alpha power, frontal-parietal alpha connectivity, or rSO₂ and postoperative cognitive scores (21). Thus, anesthetic-depth-related indices are better considered as important features within multimodal models rather than as standalone determinants of PND risk.

Randomized evidence is heterogeneous rather than uniformly positive or negative. In CODA (921 older adults undergoing major noncardiac surgery), BIS-guided anesthesia was associated with less POD (15.6% vs. 24.1%, *p* = 0.01) and less POCD at 3 months (10.2% vs. 14.7%; adjusted OR 0.67, *p* = 0.025) (77). In the Radtke trial (1,155 analyzed patients), depth monitoring reduced POD (16.7% vs. 21.4%, *p* = 0.036) but not POCD at 7 days or 3 months (78). STRIDE randomized 200 older hip-fracture patients receiving spinal anesthesia with propofol sedation; lighter sedation did not reduce POD overall (34% vs. 39%, *p* = 0.46), although a prespecified subgroup without major comorbidity suggested possible harm from heavier sedation (79). These trials addressed different anesthetic techniques, comparators, and outcomes and should not be pooled conceptually as equivalent BIS-guided general-anesthesia studies.

ENGAGES is the largest individual trial in this area: among 1,232 older adults undergoing major surgery, EEG guidance lowered age-adjusted minimum alveolar concentration (median 0.69 vs. 0.80) and EEG suppression (median 7 vs. 13 min) but did not reduce POD (26.0% vs. 23.0%, *p* = 0.22) (25). ADAPT-2 likewise reduced EEG suppression without a statistically significant POD benefit (17% vs. 20%, *p* = 0.53) (80). Conversely, the BALANCED delirium substudy reported lower POD with a lighter BIS target of 50 than with a deeper target of 35 (19% vs. 28%; OR 0.58, *p* = 0.010) among 515 patients with analyzable delirium data (81). ENGAGES-Canada found nearly identical POD rates after cardiac surgery (18.15% vs. 18.10%) despite lower anesthetic administration and suppression in the guided group (82).

At the meta-analytic level, Sumner et al. included nine trials and 4,648 participants; the pooled estimate narrowly missed conventional statistical significance (OR 0.78, 95% CI 0.60–1.00; *p* = 0.054; I^2^ = 53%) (83). An updated 2025 meta-analysis of 12 randomized trials and 4,523 participants reported a modest reduction in POD (RR 0.81, 95% CI 0.69–0.95; I^2^ = 46%), but subgroup results varied by region and North American trials did not show benefit (84). These pooled findings support possible average benefit in selected settings, not a universal causal target or device-independent index range.

Several factors may explain why promising physiological signals have not consistently translated into better outcomes. Suppression and low pEEG values may partly identify susceptible brains rather than fully modifiable causes; usual-care clinicians may already avoid excessive anesthesia, reducing between-group separation; algorithms, drugs, surgeries, adherence, co-interventions, and delirium ascertainment differ; and an intervention aimed only at hypnotic exposure addresses one component of a multifactorial syndrome. Trial interpretation should therefore distinguish proof that guidance changes an exposure from proof that changing that exposure prevents PND.

### 4.5 Principles for constructing stage-specific multimodal prediction models

Integrating raw EEG, NIRS/cerebral oximetry, derived pEEG exposure variables, and clinical variables may support research on early risk models. Raw EEG and NIRS measure different source physiology, whereas pEEG summarizes selected frontal EEG features and should not be counted as an independent information channel. PND, however, generally involves multiple factors, including baseline cognitive reserve, inflammation, cerebral perfusion, anesthetic drugs, and surgical type. An ideal prediction model should therefore incorporate preoperative, intraoperative, and early-emergence information to generate dynamic risk assessment rather than relying solely on postoperative scales. At the preoperative level, age, educational attainment, diabetes or hyperlipidemia, prior cognitive function, MoCA/MMSE scores, sleep status (with sleep-related vulnerability considered a modifiable risk factor or mediator), and baseline EEG features may be explored as risk-stratification variables. Existing studies show that spindle power in preoperative sleep EEG is associated with preoperative cognitive status and positively correlated with intraoperative alpha power, providing a feasible approach for identifying cerebral vulnerability before surgery (54). At the intraoperative level, candidate features include alpha power reduction, increased theta connectivity, decreased SEF, burst suppression, the magnitude and duration of rSO₂ decline, dynamic HbO/HbR changes, and platform-specific pEEG trends. In high-risk settings such as cardiac surgery, cardiopulmonary bypass duration, duration of hypotension, hemoglobin level, and vasoactive drug use should also be included. Model construction must also emphasize individualized baselines. EEG spectra, absolute rSO₂ values, and pEEG responses vary markedly across patients, and direct use of a uniform threshold may lead to misclassification. Compared with single measurements, relative changes calculated from a patient's own preoperative or pre-induction baseline may better reflect perioperative brain-function imbalance. For example, the magnitude and duration of rSO₂ decline from baseline may be more informative than a fixed absolute value; similarly, changes in EEG band power relative to pre-induction values may be more stable than absolute power at a single intraoperative time point. Future models should use interpretable feature combinations whenever possible, enabling anesthesiologists to understand the source of risk rather than receiving only an opaque black-box score.

In this review, ‘preoperative EEG' denotes a reportable acquisition protocol and a prespecified feature, not simply the presence of an EEG recording. Studies should state the montage and reference, resting eyes-open/eyes-closed or sleep state, recording duration, artifact criteria, and timing before sedative premedication. Candidate variables include frontal or posterior alpha power, peak alpha frequency, relative slow-wave power, aperiodic exponent or offset, and sleep-spindle power when sleep EEG is available. The evidence is limited to pilot or small cohort studies with variable acquisition states and frequent *post hoc* feature selection (2, 21, 54). No externally validated cutoff or demonstrated incremental value beyond clinical and cognitive assessment exists; preoperative EEG is therefore an exploratory research variable, not a validated screening test.

## 5 Clinical translation: devices, algorithms, and interventional trials

### 5.1 Portable and integrated monitoring devices

EEG-NIRS combined monitoring technologies are gradually moving toward wearable, integrated, and bedside real-time applications. However, these devices should be regarded as technical platforms for acquiring objective neurophysiological and oxygenation-related signals, rather than as validated diagnostic or standalone prediction tools for PND. Their clinical value depends on whether the acquired signals can be standardized, interpreted in relation to individualized baselines, and linked to clinically meaningful outcomes. Near-infrared light therapy is a separate experimental intervention and should not be conflated with NIRS/cerebral oximetry monitoring; findings from aged-mouse PND models do not establish diagnostic value for NIRS (85).

### 5.2 Artificial intelligence for interpretable perioperative time-series modeling

As multimodal signal acquisition becomes more feasible, artificial intelligence (AI) may help integrate raw EEG, NIRS/cerebral oximetry, pEEG-derived exposure variables, vital signs, anesthetic records, and surgical events into dynamic models of perioperative brain vulnerability. The role of AI should not be framed merely as improving classification accuracy or AUC, but as identifying the physiological source of risk and linking abnormal signal patterns to actionable perioperative interventions. For example, reduced alpha activity with stable rSO₂ may indicate cortical oscillatory vulnerability or increased anesthetic sensitivity, whereas sustained rSO₂ decline with EEG slowing may suggest perfusion-metabolism mismatch. These patterns require different clinical responses and therefore should not be collapsed into an opaque risk score. Future AI frameworks should prioritize multimodal perioperative time-series modeling rather than isolated single-time-point classification. Candidate inputs may include EEG spectral dynamics, burst suppression or suppression ratio, rSO₂ trajectories, cerebral desaturation burden, MAP-rSO₂ relationships, anesthetic concentration, vasoactive drug exposure, blood loss, hemoglobin level, ventilation parameters, and perioperative events such as induction, incision, cardiopulmonary bypass, emergence, and PACU recovery. For clinical translation, AI models should be evaluated using discrimination, calibration, external validation, clinical utility, and interpretability. A clinically useful model should answer three questions: who is at increased risk, which physiological abnormality is driving that risk, and what monitor-guided intervention could plausibly modify it. Therefore, model outputs should be presented as interpretable risk-stratification or decision-support information rather than as autonomous diagnostic conclusions.

No AI-based or multimodal PND prediction model is currently prospectively validated for routine clinical decision-making. Development studies should therefore compare performance with established clinical predictors, use independent external validation, and prespecify how an output would change care rather than presenting classification accuracy alone. Disease-specific quantitative EEG nomogram studies illustrate technical feasibility, but population-specific models cannot be assumed to predict PND (86).

### 5.3 Candidate signal-guided interventions and validation of clinical utility

Multimodal neuromonitoring can serve as an objective platform for evaluating perioperative brain-protection strategies, but the current evidence does not yet support a validated monitor-guided treatment algorithm for preventing PND. Overall, the translational goal of raw EEG and NIRS/cerebral oximetry, supplemented by pEEG exposure context, should be individualized brain-vulnerability phenotyping and mechanism-informed research. At present, these monitoring layers are candidate markers or decision-support inputs, not a validated standalone prediction system. These candidate actions should not be interpreted as established standards of care. The critical unanswered question is whether correcting these signal abnormalities—rather than merely detecting them—can reduce POD, DNR, or longer-term postoperative neurocognitive disorder in adequately powered randomized trials. Prospective protocols such as POEGEA also illustrate the need to prespecify the monitor-guided exposure and patient-centered neurocognitive endpoint before efficacy can be inferred (87).

For current practice, two levels of action should be distinguished. Evidence-supported care includes confirming signal quality; avoiding clearly excessive anesthetic administration when consistent with the clinical context and raw EEG; correcting conventional derangements such as hypoxemia, hypotension, abnormal ventilation, anemia, low cardiac output, or adverse head position; and applying multicomponent delirium-prevention measures (16). By contrast, titration to a specific alpha power, SEF, EEG-NIRS coupling value, rSO₂ threshold, or multimodal risk score remains hypothesis-generating. Table 3 therefore separates reasonable responses to reversible physiology from PND-specific claims that have not been validated.

A falling alpha signal should first prompt review of electrodes, artifact, the waveform or spectrogram, age, anesthetic agent and dose, temperature, and neuromuscular activity; no alpha-power target is validated. A sustained rSO₂ decline should prompt verification of probe position and the baseline, followed by assessment of SpO₂, blood pressure, PaCO₂, hemoglobin, cardiac output, head position, and surgical context; no universal rSO₂ threshold has been shown to prevent PND. Burst suppression should be confirmed on raw EEG and should prompt review of anesthetic exposure and other reversible causes when clinically appropriate, without compromising amnesia, immobility, or surgical conditions.

## 6 Closed-Loop clinical pathway for perioperative brain protection

Current studies of EEG-NIRS combined biomarkers have laid the groundwork for prediction models based on multimodal features. Subsequent clinical translation should focus on three issues: first, improving standardized acquisition procedures and artifact-processing methods; second, validating key indices across different surgical populations, anesthetic agents, and baseline cognitive states; and third, integrating multimodal indices with clinical variables to form an interpretable and actionable risk-stratification system. Traditional perioperative management relies mainly on vital signs and empirical anesthetic dosing, while objective assessment of brain-function status remains insufficient. Raw EEG and NIRS/cerebral oximetry can provide complementary functional information, while pEEG supplies a pragmatic exposure trend; together they may support intraoperative interpretation but do not constitute a validated PND prediction system. On the one hand, real-time monitoring may support individualized anesthetic management and reduce risks associated with insufficient or excessive anesthesia, including awareness and neurotoxic exposure (49). On the other hand, preoperative baseline EEG features and rSO₂ levels may help identify patients with cerebral vulnerability and support early intervention in high-risk populations (2). However, this monitoring framework cannot currently be recommended as a routine PND-prevention standard for all surgical patients. Its widespread implementation is limited by nonuniform device interfaces, complex intraoperative artifacts, NIRS susceptibility to scalp blood flow and probe position, insufficient BIS sensitivity outside frontal regions, and lack of unified interventional thresholds. In the future, real-time analysis systems incorporating AI may enable automated risk stratification and drive perioperative brain-health management toward greater precision and dynamism (88–90). Figure 3 summarizes this research-oriented pathway from multimodal feature acquisition to risk profiling and evidence-bounded clinical decision support.

**Figure 3:**
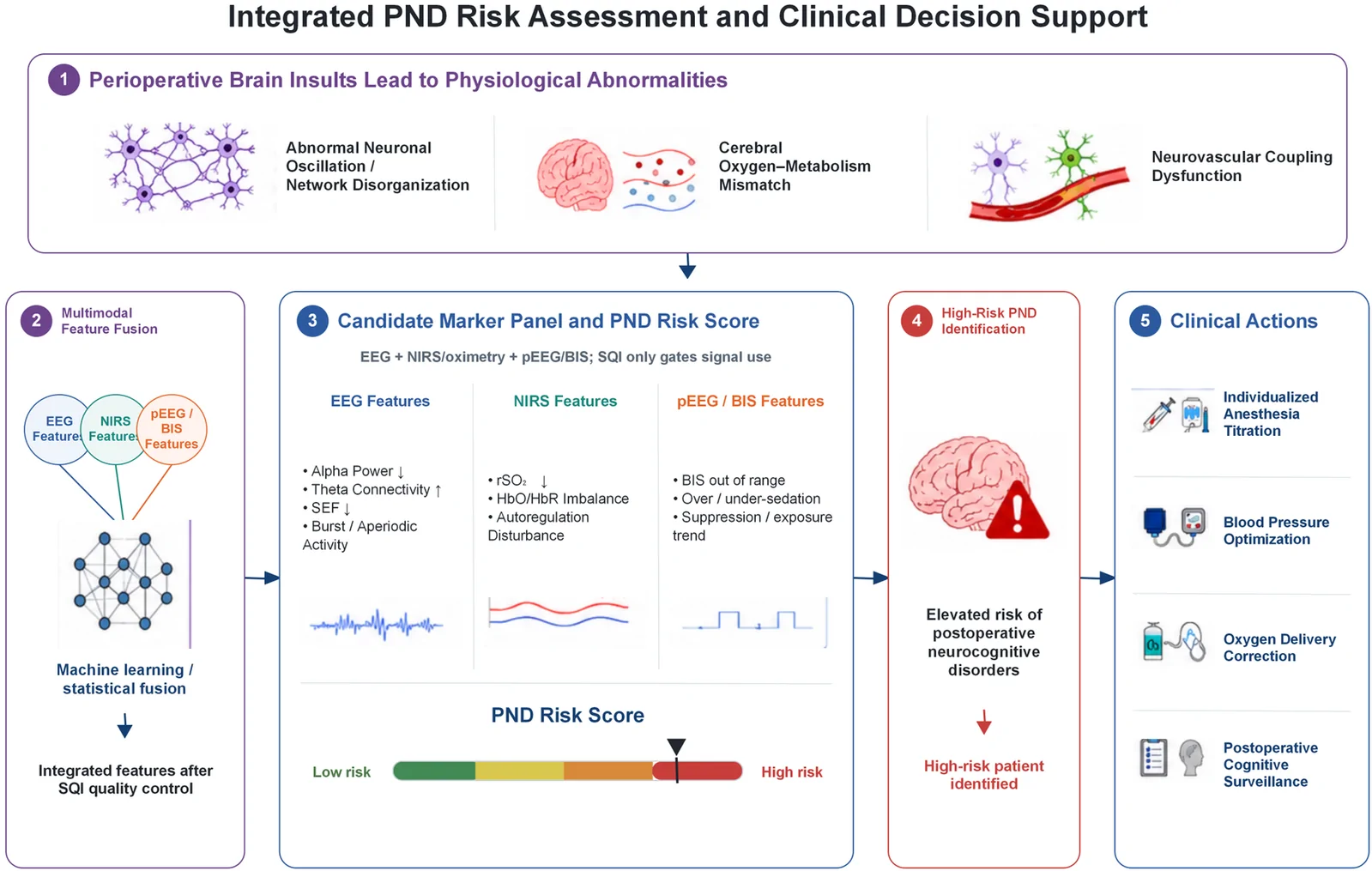
Research-oriented framework for integrated PND risk assessment with SQI-based signal-quality control.

Perioperative brain insults may generate abnormalities in neuronal oscillations, cerebral oxygen–metabolism balance, and neurovascular coupling. Synchronously acquired EEG, NIRS/cerebral oximetry, processed EEG/BIS, and clinical variables can be combined into candidate features and a research-stage PND risk score. The Signal Quality Index (SQI) is used only as a signal-quality gate: poor-quality pEEG/BIS segments are excluded from feature extraction and do not contribute to the risk score. A high-risk profile may support individualized review of anesthetic exposure, blood pressure and cerebral perfusion, oxygen delivery, and postoperative cognitive surveillance. The framework is intended to organize hypothesis generation and prospective validation; it does not represent a validated prediction model or a prespecified treatment algorithm.

## 7 Limitations of this narrative review and future directions

This review has inherent limitations as a narrative review. First, it was not registered and did not include systematic screening by two independent reviewers or formal risk-of-bias assessment; therefore, it cannot substitute for a systematic review or meta-analysis. Second, diagnostic criteria, assessment tools, and follow-up periods for PND, POD, DNR, and POCD vary substantially across studies, making evidence synthesis and index comparison difficult. Third, EEG spectral features, NIRS cerebral oxygenation metrics, and BIS values are all affected by age, anesthetic drugs, temperature, hemodynamics, surgical type, and preoperative cognitive status, and unified absolute thresholds remain difficult to establish. Fourth, most current multimodal studies are single-center, small-sample, observational, or methodological studies; prospective validation and interventional trials specifically in PND populations remain insufficient. Future research should use standardized signal acquisition and artifact-processing procedures, prespecify analytic windows and primary outcomes, report model discrimination, calibration, and decision curves, and further determine whether monitor-guided anesthetic-depth adjustment, cerebral perfusion optimization, oxygenation and ventilation correction, and anti-delirium strategies can truly improve neurocognitive outcomes.

Priority directions include continuous postoperative or wearable EEG, portable oxygenation monitoring, digital recovery markers, combinations of EEG with blood biomarkers, and transparent machine-learning models. These approaches require prespecified outcomes, multicenter external validation, calibration and decision-curve reporting, and trials that link a defined signal abnormality to a feasible intervention and a patient-centered neurocognitive endpoint.

## 8 Conclusions

PND results from the interaction between baseline cerebral vulnerability and multiple perioperative stressors, and a single monitoring index cannot adequately explain its dynamic risk. NIRS/cerebral provide information on cortical cerebral and Their integration may support a more interpretable framework for perioperative brain-vulnerability At present, EEG-NIRS monitoring supplemented by pEEG exposure trends is more appropriately regarded as a risk-stratification and research tool for high-risk patients than as a routine standard for all surgical patients. Future studies should focus on individualized baseline calibration, standardized acquisition, interpretable multimodal modeling, and closed-loop interventional validation, so that PND management can gradually shift from delayed postoperative recognition toward mechanism-informed risk stratification and precision perioperative brain protection.
